# Enhancement of Gut Microbial Homeostasis by a Post-NGP *Phocaeicola vulgatus*

**DOI:** 10.3390/nu18142355

**Published:** 2026-07-17

**Authors:** Md Sarower Hossen Shuvo, Sukyung Kim, Sujin Jo, Izaz Ahmed, Md Tareque Aziz, Youjin Yoon, Faezeh Sarafraz, Sera Oh, Gi Tae Nam, Jaehyuk Lee, Yeram Im, So Yeong Park, So Youn Gong, Min Gyu Kang, Seo Hyeon Jang, Soon Hyo Kwon, Hoonhee Seo, Ho-Yeon Song

**Affiliations:** 1 Department of Microbiology and Immunology, School of Medicine, Soonchunhyang University, Cheonan 31151, Chungnam, Republic of Korea; 2 K-Microbiome Institute, 813-26 Yisunsin-Daero, Asan 31462, Chungnam, Republic of Korea; 3 Next-Generation Microbiome Training Center, Soonchunhyang University, 22 Soonchunhyang-Ro, Sinchang, Asan 31538, Chungnam, Republic of Korea; 4 Division of Nephrology, Department of Internal Medicine, Soonchunhyang University Hospital, Soonchunhyang University College of Medicine, Seoul 04401, Republic of Korea

**Keywords:** post-next-generation probiotics (Post-NGP), *Phocaeicola vulgatus* PMC94, gut microbial homeostasis, human gut microbiome simulator

## Abstract

Background: Humans have long consumed lactic acid bacteria-based fermented foods, and this empirical experience has led to the development of probiotic-based functional foods and therapeutics. However, conventional development strategies have largely focused on commonly used probiotic strains to prioritize development efficiency and safety, resulting in limited functional innovation. Although research on next-generation probiotics (NGPs) has expanded in recent years, there is an increasing need for post-next-generation probiotic (Post-NGP) strategies that address subsequent stages of microbiome modulation. Methods: In this study, *Phocaeicola vulgatus* PMC94 was isolated and characterized as a Post-NGP candidate, and its effects on gut microbiome balance were evaluated using ex vivo human gut microbiota culture (ex vivo HGMC). Results: Dysbiosis induced by commonly encountered therapeutic agents was significantly alleviated by co-administration of PMC94. This restorative effect on gut microbiome imbalance was more pronounced than that observed with conventional probiotic strains. To elucidate the mechanistic basis underlying these effects, additional analyses were conducted using a human gut microbiome simulator (HGMS). PMC94 selectively suppressed *Proteobacteria* while promoting balanced proliferation of *Bacteroidetes* and *Firmicutes*, thereby restoring gut microbial homeostasis. This pattern of microbiome modulation was consistently supported by in vivo mouse experiments. Furthermore, these changes were associated with increased production of short-chain fatty acids (SCFAs), as well as immune modulation and reinforcement of gut barrier function. The safety of PMC94 was confirmed through a 2-week repeated-dose toxicity study. Conclusions: Collectively, these findings demonstrate that *P. vulgatus* PMC94 is a promising Post-NGP candidate capable of restoring and strengthening gut microbial homeostasis.

## 1. Introduction

The human body harbors a complex microbial ecosystem composed of thousands of microbial species, which collectively play fundamental roles in host metabolism, immune regulation, and the development and progression of disease [1]. Over the past decades, accumulating evidence has established the gut microbiome as a critical determinant of human health and disease [2]. Despite this remarkable microbial diversity, the development of microbiome-based therapeutics and functional probiotics has largely focused on a limited group of well-known, easily cultivable microorganisms, particularly traditional probiotic strains such as lactobacilli and bifidobacteria [3,4]. Although these conventional probiotics are generally regarded as safe and capable of conferring measurable health benefits, their effects are often modest, transient, and insufficient to address complex dysbiotic conditions [3,5]. These limitations suggest that the therapeutic potential of the gut microbiome remains far from fully explored and that functionally important microorganisms with greater therapeutic potential may remain undiscovered.

To overcome these limitations, increasing attention has been directed toward next-generation probiotics (NGPs), which consist of commensal microorganisms associated with health or disease states [6]. Representative candidates, such as *Akkermansia* and *Faecalibacterium,* have been proposed as promising therapeutic microbes due to their functional roles in the gut ecosystem [7]. However, NGPs still represent only a small fraction of the vast microbial diversity present in the human gut microbiome. They therefore cannot be considered a universal solution to the limitations of conventional probiotics [6,8]. In many cases, candidate strains are selected primarily on the basis of correlation with host phenotypes rather than causal functional validation, and their activity can be highly context-dependent within the complex ecological interactions of the gut microbiome [9]. These considerations indicate that current probiotic and NGP-centered strategies utilize only a limited portion of the available microbial diversity and that unexplored microorganisms with greater functional potential may still exist within the gut microbiome.

Indeed, many current microbiome-based therapeutic development strategies continue to rely on microorganisms that are already known, frequently reported, or relatively easy to cultivate [10]. Such approaches may introduce structural bias in strain discovery and limit the identification of functionally superior microbial candidates [11]. Furthermore, many development pipelines still treat candidate strains as incremental extensions of existing probiotic categories rather than as members of a qualitatively distinct class of live biotherapeutic agents [11,12]. Given the immense taxonomic diversity and functional capacity of the gut microbiome, expanding existing probiotic lineages alone may not be sufficient to harness the microbiome’s therapeutic potential fully [13]. These limitations highlight the need for a more systematic conceptual framework for discovering and prioritizing functionally meaningful microbial candidates.

In this context, we introduce the concept of post-next-generation probiotics (Post-NGP). Post-NGP does not simply refer to taxonomically novel microorganisms but rather represents a systematic discovery framework for identifying microbiome-derived therapeutic candidates from the vast diversity of the gut microbiome. This concept moves beyond empirical strain selection and restricted taxonomic exploration by integrating multiple analytical layers, including culturomics-based strain isolation, genomic characterization, function-oriented screening, and physiological validation. Such a framework enables a more structured exploration of microbial diversity while considering the ecological complexity of the gut microbiome, thereby facilitating the identification of previously overlooked microbial candidates with therapeutic potential.

Based on this conceptual framework, the present study applied an integrated strategy combining culturomics-driven strain discovery with multilayer functional evaluation to identify Post-NGP candidates. Through this approach, *Phocaeicola vulgatus* strain PMC94, isolated from the human gut microbiota, was identified as a potential Post-NGP candidate. Using genome-based characterization, ex vivo human gut microbiome culture systems, a human gut microbiome simulator, and in vivo validation in BALB/c mice, we evaluated PMC94’s ability to modulate the gut microbiome and restore microbial balance selectively. These findings demonstrate that a systematic discovery strategy can identify microbiome-derived therapeutic candidates beyond conventional probiotics and currently recognized NGPs.

## 2. Materials and Methods

### 2.1. Collection and Anaerobic Processing of Human Fecal Samples

The study aimed to isolate a Post-NGP bacterium with a high likelihood of outperforming existing NGP candidates in probiotic activity. Fresh stool samples were collected from 17 healthy adult volunteers recruited from Soonchunhyang University Hospital, Seoul, Republic of Korea. Volunteers had no history of gastrointestinal disease and had not received antibiotics or probiotic supplements within the previous three months. The bacterial isolation process adhered to previously established protocols [14,15]. All procedures were conducted in accordance with the Declaration of Helsinki and approved by the Institutional Review Board (IRB) of Human Research at Soonchunhyang University Seoul Hospital (SCH 2019-12-004) [14]. To preserve obligate anaerobic commensals, stool samples were placed in sterile airtight containers and transferred to the laboratory immediately after collection. Upon arrival, samples were promptly introduced into an anaerobic chamber (The Baker Co., Sanford, ME, USA). All sample handling, dilution, and enrichment procedures were performed under strictly anaerobic conditions (37 °C temperature, 85% relative humidity, 0% O_2_, and 5.0% CO_2_) using chopped meat media (Kisan Bio, Seoul, Republic of Korea).

### 2.2. Culturomics-Based Isolation of Gut Microbial Strains

A modified culturomics-based strategy, combined with 16S rRNA gene sequencing, was used to isolate diverse gut microbial strains from healthy human stool samples. Briefly, stool samples were homogenized in modified phosphate-buffered saline (PBS, pH 7.2) supplemented with L-cysteine (Sigma-Aldrich, St. Louis, MO, USA) as a reducing agent and serially diluted up to 10^−5^. Pre-enrichment was performed by inoculating diluted samples (1%, *v*/*v*) into chopped meat media (Kisan Bio, Seoul, Republic of Korea) and incubating anaerobically at 37 °C for 48 h. The enriched cultures were subsequently subjected to serial dilution (up to 10^−8^) and plated onto a variety of selective and non-selective media, including Rogosa SL agar (BD Biosciences, Franklin Lakes, NJ, USA), MRS agar (BD Biosciences, Franklin Lakes, NJ, USA), modified MRS agar supplemented with bromocresol purple and L-cysteine (Sigma-Aldrich, St. Louis, MO, USA), Columbia blood agar (KisanBio, Seoul, Republic of Korea), Brucella anaerobic agar (BD Biosciences, Franklin Lakes, NJ, USA), Reinforced Clostridial agar (Oxoid, Hampshire, UK), and Bifidobacterium agar (BD Biosciences, Franklin Lakes, NJ, USA). All plates were incubated in an anaerobic chamber (The Baker Co.) at 37 °C with 85% relative humidity, 0% O_2_, and 5.0% CO_2_ for 48 h, with extended incubation up to 7 days for slow-growing species. Approximately 20–30 distinct colonies were selected from each culture plate based on morphological characteristics, including size, shape, and pigmentation, and sub-cultured repeatedly to obtain pure isolates. Finally, purified isolates were preserved at −80 °C in 30% (*v*/*v*) glycerol for subsequent analyses.

### 2.3. Bacterial Identification Using Capillary Electrophoresis-Based Sequencing

The primary taxonomic identification of the strain was conducted using 16S rRNA gene sequencing, as previously described [16,17]. Genomic DNA was prepared, and the DNA was amplified through PCR with universal bacterial primers 27F (5′-AGA GTT TGA TCC TGG CTC AG-3) and 1492R (5′-GGT TAC CTT GTT ACG ACT T-3) [18]. The resulting amplicons were purified and sequenced on an ABI PRISM 3730XL DNA Analyzer (Applied Biosystems, Foster City, CA, USA). All sequencing procedures were performed by Biofact (Daejeon, Korea). The acquired sequence data were analyzed by comparing them against the reference sequence in the NCBI GenBank database using the BLAST (version 2.13.0) algorithm to determine species-level identity.

### 2.4. Whole-Genome Sequencing of the Candidate Strain

The candidate strain was subjected to a comprehensive whole-genome analysis, including sequencing, genome assembly, annotation, and comparative genomic analysis according to the protocol described in a previously published study [19]. The gDNA was extracted using a QIAamp DNA Mini Kit (Qiagen, Hilden, Germany) after washing the bacterial culture with PBS. Chunlab developed PacBio libraries and performed whole-genome sequencing. The genomic DNA was purified using a g-tube (Covaris, Woburn, MA, USA), which separates it into 10 kb fragments. After fixing the ends, SMRTbell adapters were ligated to the blunt ends using the SMRTbell Template Prep Kit 1.0 (PacBio, Menlo Park, CA, USA). The library was then sequenced on an 8-well SMART Cell v3 using the PacBio RSII with PacBio P6C4 chemistry. PacBio SMRT Analysis 2.3.0 was used to assemble the PacBio sequencing data with the HGAP2 procedure. Finally, the genome was circularized using Circlator 1.4.0 (Sanger Institute, Hinxton, UK). Genome assembly statistics, including genome size and N50, were calculated from the assembled contigs, while genome completeness and contamination were estimated using CheckM [20], based on the recovery of lineage-specific marker genes. Prodigal 2.6.2 [21] was utilized to identify protein-coding sequences (CDSs), which were then analyzed for their roles in orthologous groups using EggNOG [http://eggnog5.embl.de\, accessed on 13 January 2026]. tRNAscan-SE 1.3.1 was used to detect genes encoding tRNAs [22]. The search for covariance models of rRNAs and other noncoding RNAs was conducted using the Rfam 12.0 database [23]. To compare prokaryotic genome sequences, the Average Nucleotide Identity (ANI) calculator based on the OrthoANIu algorithm was used [24].

### 2.5. Cultivation and Preparation of the Post-NGP Candidate

Chopped meat broth (Kisan Bio) was used to grow the Post-NGP. The inoculated culture (1%) was incubated for 48 h at 37 °C under strict anaerobic conditions using an anaerobic chamber (The Baker Co.) maintained at 85% relative humidity, 0% O_2_, and 5.0% CO_2_ throughout incubation; O_2_ levels within the chamber were continuously monitored via the chamber’s built-in oxygen sensor, and an anaerobic indicator strip was additionally included to confirm anaerobiosis was maintained throughout the incubation period. After incubation, the bacterial cultures were standardized to an optical density of 1.0 at 600 nm (OD600) using a spectrophotometer (DR 1900, Hach, Loveland, CO, USA). Cells were harvested by centrifugation at 3000× *g* for 30 min (Hanil Scientific, Gimpo, Republic of Korea) and washed with sterile 0.85% NaCl solution to eliminate residual media components that could interfere with downstream analyses. The resulting cell pellet was resuspended in 1 mL of 0.85% NaCl solution and used in subsequent experiments.

### 2.6. Experimental Setup of a Dynamic Gut Microbiome Simulation System

The impact of the candidate strain on gut microbial dynamics was studied using a Human Gut Microbiome Simulation (HGMS) system based on the validated dynamic in vitro model of the human gastrointestinal tract, SHIME^®^ (ProDigest, Ghent, Belgium) [25,26]. The HGMS consists of five interconnected bioreactors that simulate different regions of the gut: the stomach (ST), small intestine (SI), ascending colon (AC), transverse colon (TC), and descending colon (DC). The ST and SI compartments operate in a fill-and-draw mode to replicate physiological feeding and digestion. Specifically, 140 mL of standardized nutritional media is added to the ST vessels three times a day. The standardized nutritional media consisted of carbohydrates (e.g., starch, pectin, and glucose), protein sources (e.g., peptone and yeast extract), lipids, minerals, and vitamins, formulated to mimic the average human diet and support microbial growth. In comparison, 60 mL of pancreatic-bile solution is added to the SI vessels at designated intervals. The pancreatic-bile solution contained pancreatin (as a source of digestive enzymes) and bile salts to simulate small intestinal digestion conditions. The contents of these compartments are emptied after predetermined retention periods to mimic gastric emptying and intestinal transit. A circulating water bath maintains all reactors at 37 °C, and the pH levels of the colon compartments (AC, TC, and DC) are continuously monitored and automatically adjusted to physiological levels using 0.5 M NaOH or HCl. Each vessel is stirred continuously at 300 rpm throughout the experiment to ensure steady mixing and maintain microbial activity. To perform a comparative analysis, one HGMS unit was kept as a control (untreated), and the parallel unit was supplemented daily with the candidate strain (1 × 10^10^ CFU). After stabilization and treatment within the HGMS, microbial samples were aseptically collected from the colon compartments and transferred to the ex vivo multi-treatment human gut microbiota culture (ex vivo HGMC) model. The ex vivo HGMC served as a secondary culture platform to evaluate the responses of the established microbial communities to multiple supplementation strategies under identical baseline conditions. The samples were incubated for five days at 37 °C under constant agitation in a shaker (Biofree, Seoul, Republic of Korea) to simulate intestinal peristalsis and ensure uniform exposure of the microbes to the test treatments. The experimental parameters included treatment with the candidate, several traditional probiotic bacteria, and clinically relevant therapeutic agents.

### 2.7. Tested Functional Ingredients, Pharmaceutical Agents, and Probiotic Strains

Based on functional classification, we tested five types of therapeutic agents categorized according to their primary biological roles: (1) anti-inflammatory agents, including loxoprofen sodium (Dongwha Pharmaceutical Co., Ltd., Seoul, Republic of Korea) and acetylsalicylic acid (aspirin; Bayer Korea Ltd., Seoul, Republic of Korea); (2) metabolic modulators related to bile acid and lipid metabolism, including ursodeoxycholic acid (Ursa^®^; Daewoong Pharmaceutical Co., Ltd., Seoul, Republic of Korea) and omega-3 fatty acids containing eicosapentaenoic acid (EPA) and docosahexaenoic acid (DHA) (Daewoong Pharmaceutical Co., Ltd., Seoul, Republic of Korea); (3) antioxidant and phytochemicals, including ascorbic acid (vitamin C; Yuhan Corporation, Seoul, Republic of Korea), silymarin (milk thistle extract; Kolmar Korea Co., Ltd., Sejong, Republic of Korea), propolis extract (Ildong Pharmaceutical Co., Ltd., Seoul, Republic of Korea), and an antioxidant combination supplement (Beada^®^; Kolmar Korea Co., Ltd., Sejong, Republic of Korea); (4) physicochemical stressors, including potassium citrate combined with potassium chloride (Potacin Duo^®^; Hanmi Pharmaceutical Co., Ltd., Hwaseong, Republic of Korea) and methylsulfonylmethane (MSM; Newtree Co., Ltd., Seoul, Republic of Korea); and (5) neuroactive drugs, including tramadol hydrochloride (Tramol^®^; Yuhan Corporation, Seoul, Republic of Korea) and eperisone hydrochloride sustained-release formulation (Eperil-M SR^®^; Hanmi Pharmaceutical Co., Ltd., Hwaseong, Republic of Korea). Each compound was tested individually to evaluate its direct impact on microbial community composition and identify drug-induced dysbiosis patterns. Conventional probiotic strains were isolated from traditional fermented foods and used in this study. The strains included *Latilactobacillus curvatus*, *Latilactobacillus sakei*, *Pediococcus pentosaceus*, *Lactococcus lactis*, and *Bacillus velezensis*. Taxonomic identification was performed using 16S rRNA gene sequencing (Table S1). All probiotic strains were tested at 5 × 10^8^ CFU/mL, and each compound was applied at 1 mg/mL. The concentration was selected as a standardized screening dose to enable comparative evaluation of different compounds on the gut microbial community under the in vitro experimental conditions. Prior to use, all pharmaceutical agents were completely dissolved in the appropriate sterile solvent according to the manufacturer’s recommendations to ensure complete solubility and consistent dosing across treatments.

### 2.8. NGS-Based Fecal Metagenomic Analysis

Metagenomic analysis was performed based on 16S rRNA gene sequencing, following the procedures developed by our group [27]. Collected samples underwent total microbial DNA extraction using the QIAamp DNA Mini Kit (Qiagen), and DNA concentrations were quantified with a Qubit 4 fluorometer (Thermo Fisher Scientific, Waltham, MA, USA). The integrity and quality of the DNA were confirmed by running samples on a 0.8% agarose gel, and purified DNA samples were stored at −20 °C until use. Region-specific primers were utilized (Forward: TCG TCG GCA GCG TCA GAT GTG TAT AAG AGA CAG CCT AGG GGN GGC WGC AG; Reverse: GTC TCG TGG GCT CGG AGA TGT GTA TAA GAG ACA GGA CTA CHV GGG TAT CTA ATCC) to amplify the V4 hypervariable region of the 16S rRNA gene. PCR amplification was performed using KAPA HiFi HotStart ReadyMix, followed by purification with AMPure XP magnetic beads (Beckman Coulter, Brea, CA, USA) [28,29]. The Nextera XT DNA Library Preparation Kit (Illumina, San Diego, CA, USA) was employed for sequencing on the Illumina iSeq100 platform (Illumina, San Diego, CA, USA). Raw sequencing reads were analyzed using Quantitative Insights into Microbial Ecology 2 (QIIME 2) (version 2022.2) [30]. The DADA2 algorithm was used to produce quality-filtered, denoised, and dereplicated sequences, generating amplicon sequence variants (ASVs) at a single-nucleotide resolution [31]. Prior to downstream analyses, feature tables were normalized to account for differences in sequencing depth across samples. Specifically, alpha and beta diversity analyses were performed on rarefied data using an even sequencing depth determined based on the sampling depth distribution [32]. Representative ASV sequences were assigned taxonomic categories using a naive Bayes classifier based on a reference database and further curated with the Human Microbiome Database to refine the classifications [27,33]. A Bayesian classification approach with a minimum confidence level of 97% was implemented to ensure accurate and reliable taxonomic assignments. Alpha diversity measures, including Chao1, Fisher, Simpson, and Shannon indices, were calculated to assess microbial richness and diversity [34,35,36,37]. To compare community composition among samples, Bray–Curtis dissimilarity and Jensen–Shannon divergence were employed to evaluate beta diversity [38,39,40]. Statistical assessment of beta diversity differences was conducted using the permutational multivariate analysis of variance (PERMANOVA) [41]. LEfSe (version 1.1.01) was utilized to identify taxonomic differences between groups after normalization to relative abundance [42].

### 2.9. Analysis of Short-Chain Fatty Acids by Gas Chromatography-Mass Spectrometry

Short-chain fatty acid (SCFA) analysis was conducted as previously described [43]. Simulated gut ecosystem samples were collected on days 0, 4, and 7 during continuous supplementation with the candidate strain, and SCFA concentrations were measured. In each experiment, 2.5 g of sodium chloride was added to 1 mL of HGMS effluent in sealed headspace vials, along with 1 mL of 2% sulfuric acid. The samples were analyzed using a TurboMatrix Headspace Sampler connected to a gas chromatography-mass spectrometry (GC-MS) system. A PerkinElmer Clarus 690 GC system was equipped with a Clarus SQ8 mass spectrometer and an Elite-FFAP capillary column (30 m length, 0.25 mm inner diameter, and 0.25 µm film thickness) for SCFA separation. The injector temperature was set to 250 °C, with split injection (5:1) and an injection volume of 0.16 mL. Helium served as the carrier gas, maintained at a fixed flow rate of 16 mL/min. The oven temperature was gradually increased to 200 °C at 5 °C/min. The ion source temperature was adjusted to 250 °C, and mass spectra were obtained in full-scan mode. Quantification was achieved using external calibration curves generated from standard solutions of acetic, propionic, butyric, and valeric acids (1–100 mg/L). SCFA identification in experimental samples was performed by matching retention times and mass spectral profiles with the corresponding standards.

### 2.10. Quantitative Analysis of Gene Expression in Intestinal Tissues

Gene expression analysis was conducted according to the established protocol [44]. Quantitative real-time PCR (qRT-PCR) was used to measure gene expression levels to assess the impact of the candidate strain on intestinal barrier integrity and immune control. The strain was administered orally to six-week-old female BALB/c mice at a dose of 5 × 10^8^ CFU per mouse per day for a duration of two weeks. Intestinal tissues were collected, and total RNA was extracted at the end of the treatment using the RNeasy Mini Kit (Qiagen, Hilden, Germany) according to the manufacturer’s instructions. The concentration of RNA was measured with a Qubit Fluorometer (Invitrogen, Carlsbad, CA, USA) and the Qubit RNA Assay Kit (Thermo Fisher Scientific, Waltham, MA, USA). Purified RNA was then reverse transcribed using a cDNA synthesis kit (Bio-Rad, USA). Quantitative PCR amplification was performed with SYBR Green Supermix (Bio-Rad, Hercules, CA, USA) using a CFX96 Real-Time PCR Detection System (Bio-Rad, USA). The analysis focused on genes associated with tight junction integrity in the intestine, including zonula occludens-1 (ZO-1), claudin, occludin, and the anti-inflammatory cytokine interleukin-10 (IL-10). Normalization was performed using the housekeeping gene (*Gapdh*). The relative gene expression ratios were determined using the comparative Ct (2^−ΔΔCt^) method, as previously described [45]. Primer sequences to be analyzed in qRT-PCR are listed in Supplementary Table S2.

### 2.11. In Vivo Safety Evaluation of the Post-NGP Candidate

To assess the safety of the candidate strain in animals, an acute oral toxicity study was conducted in accordance with a previously published report [46]. In this repeated-dose oral toxicity experiment, six-week-old female BALB/c mice were randomly assigned to two groups (*n* = 5 per group). One group received the strain orally at a dose of 5 × 10^8^ CFU per mouse daily for two weeks, while the other group received distilled water. The animals were kept under standardized housing conditions and had free access to food and water. Throughout the experiment, the mice were monitored daily for signs of toxicity, mortality, and changes in body weight. Clinical endpoints included general appearance, posture, locomotor activity, grooming behavior, food and water intake, fur condition, diarrhea, lethargy, and any other abnormal behavioral or physical changes. Toxicity was defined by the occurrence of mortality, severe clinical signs, marked body weight loss, or persistent abnormal behavior. All animal experiments were conducted in accordance with institutional and national animal-testing standards, and the study was approved by the Institutional Animal Care and Use Committee (IACUC) of Soonchunhyang University (Approval No. SCH25-0029).

## 3. Results

### 3.1. Culturomics-Based Discovery of Post-NGP Candidates and Cell Banking

**Figure 1:**
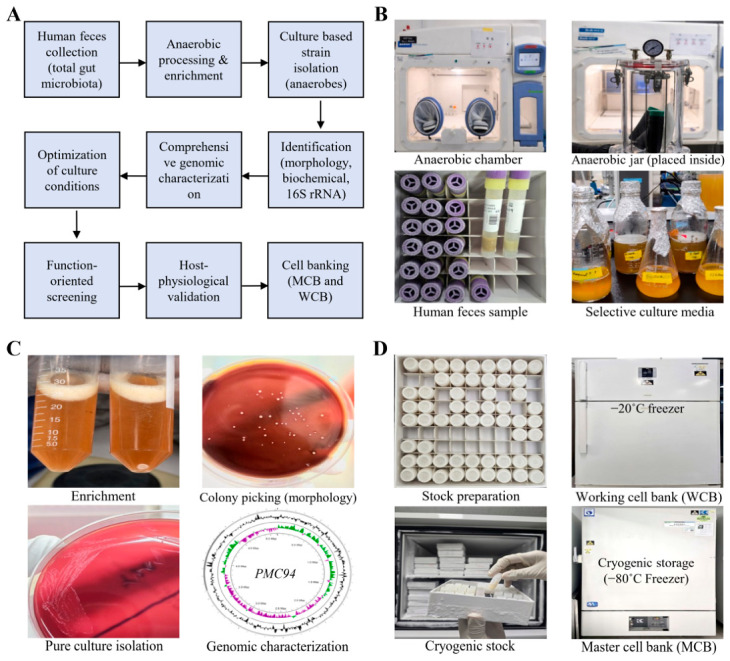
**Experimental workflow for culture-based discovery of Post-NGP candidates.** (**A**) A stepwise experimental protocol outlines anaerobic isolation, identification, and screening of gut-derived microbial strains for systematic discovery of Post-NGP candidates. (**B**) Anaerobic culture environment and materials were used for strain isolation, including an anaerobic chamber, human fecal samples, and selective culture media that support diverse gut microorganisms. (**C**) Enrichment and pure culture isolation were performed through morphology-based colony selection, followed by systematic screening and validation of candidate strains using genomic and functional characterization. (**D**) Bacterial stocks were prepared and preserved as aliquot cultures for long-term storage, with the Working Cell Bank (WCB) stored at −20 °C and the Master Cell Bank (MCB) preserved at −80 °C to ensure strain maintenance and reproducibility.

A culture-based workflow, combined with anaerobic processing and genomic identification, was established to isolate and characterize functionally relevant Post-NGP candidates systematically (Figure 1). A stepwise anaerobic culture-based isolation strategy for gut bacterial strains from human feces is outlined, followed by taxonomic identification and functional validation within an integrated experimental framework (Figure 1A). Initially, the total gut microbiota obtained from human feces underwent anaerobic processing and enrichment to selectively promote the growth of obligate anaerobes (Figure 1B). The experimental setup incorporated a controlled anaerobic chamber and anaerobic jar system to maintain oxygen-free conditions required for strict anaerobe cultivation. Selective culture media supported the differential growth of gut bacterial populations derived from fecal samples. Distinct colonies with varied morphology were obtained and purified by repeated subculturing (Figure 1C). Genomic characterization and host-physiological validation demonstrated the suitability of the isolated strain for downstream biological applications. For reproducibility and long-term preservation, standardized cell banking procedures were performed, including preparation of master cell bank (MCB) and working cell bank (WCB) stocks (Figure 1D). Cryogenic storage at −80 °C maintained strain viability and genetic stability. This structured workflow enabled successful isolation, identification, functional assessment, and preservation of Post-NGP strains for subsequent mechanistic and therapeutic studies.

### 3.2. Genomic and Taxonomic Characterization of P. vulgatus PMC94

**Figure 2:**
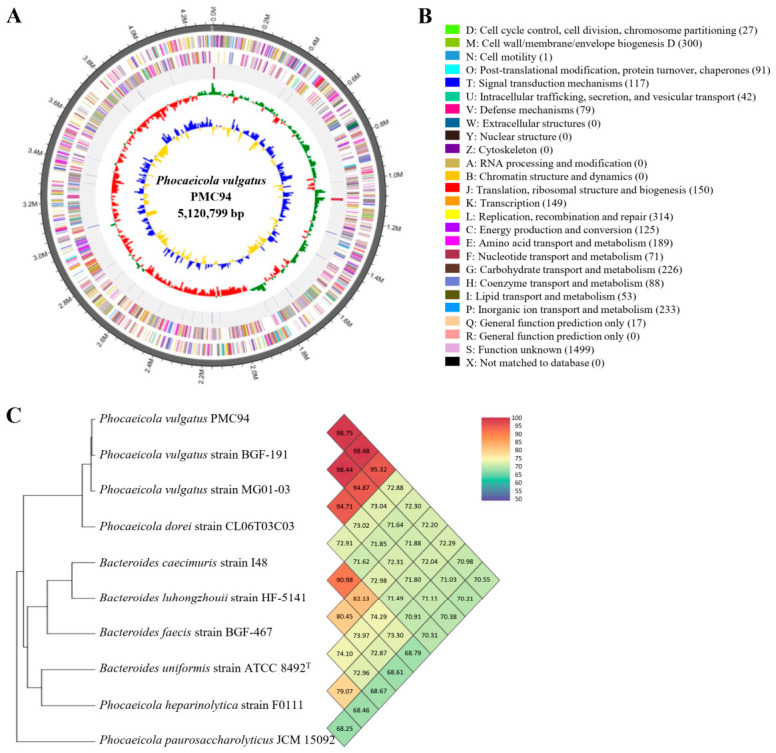
**High-throughput genome sequencing of *Phocaeicola vulgatus* PMC94.** (**A**) A circular genome map illustrating the complete chromosome of the *P. vulgatus* PMC94 strain is shown. Antisense and sense strands (colored according to COG categories) and RNA genes (red, tRNA; blue, rRNA) are shown from the outer periphery to the center. Inner circles show GC skew, with yellow and blue indicating positive and negative values, respectively, and GC content shown in red and green. This genome map was visualized using CL Genomics. (**B**) The relative abundance of clusters of orthologous groups (COG) functional categories of genes is presented separately. (**C**) The phylogenomic tree and OrthoANI results were calculated using available genomes of *Phocaeicola* species. The results between two strains are given at the junction point of the diagonals departing from each strain; for example, the OrthoANI value between *P. vulgatus* PMC94 and *P. vulgatus* strain BGF-191 is 98.75%.

The Post-NGP candidate *Phocaeicola vulgatus* was initially identified by 16S rRNA gene sequencing, in which BLAST analysis showed the highest similarity to *P. vulgatus* (99% identity; 1435/1440 bp) compared with NCBI reference sequences (Table 1). For further confirmation and comprehensive functional characterization, whole-genome sequencing was subsequently performed (Figure 2 and Table 2). The genome of *P. vulgatus* PMC94 comprises a single circular chromosome of 5,120,799 bp, with a GC content of 42.2%. In total, 4269 coding sequences were identified, along with 21 rRNA genes and 81 tRNA genes (Figure 2A). Functional annotation based on the Clusters of Orthologous Groups (COG) indicated a broad distribution of genes across the genome, with genes related to metabolism, information processing, and cellular functions (Figure 2B). A comparative genomic analysis using the Orthologous Average Nucleotide Identity (OrthoANI) method showed that PMC94 has the highest nucleotide similarity to reference strains of *P. vulgatus*, thereby confirming its taxonomic classification within this species (Figure 2C). Although PMC94 is grouped within the *P. vulgatus* lineage, it exhibits distinct chromosomal characteristics in terms of genome size and gene content compared to other strains, as summarized in Table 2, which supports its classification as a novel strain.

### 3.3. P. vulgatus PMC94-Driven Restoration of Gut Microbiota Homeostasis in Ex Vivo HGMC

**Figure 3:**
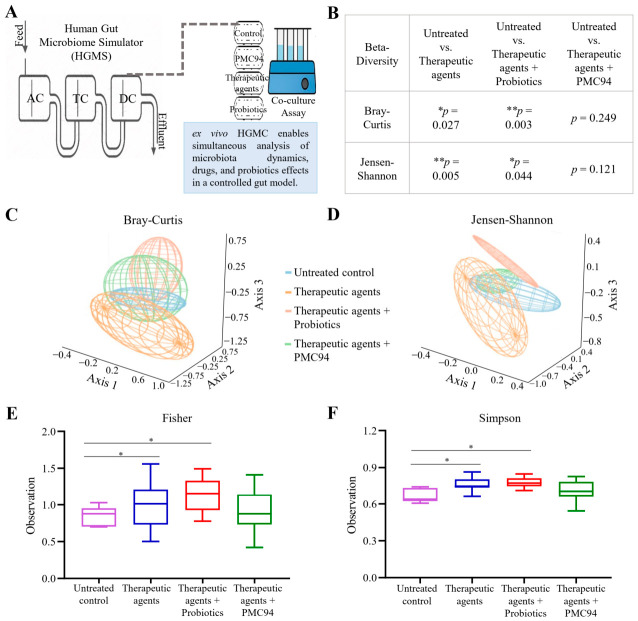
**Effects of therapeutic agents on gut microbial community structure.** The effects of therapeutic agents on gut microbial community structure and diversity were evaluated in an ex vivo HGMC system, with co-administration of conventional probiotics or post-NGP. (**A**) Schematic overview of the ex vivo HGMC system, illustrating anaerobic fermentation compartments and a downstream co-culture assay for evaluating microbial community dynamics and the effects of multiple interventions. (**B**) Beta diversity comparisons based on PERMANOVA showed significant differences (* *p* < 0.05, ** *p* < 0.01) between the untreated group and the therapeutic agents-treated group. Significant differences were also observed in the therapeutic agents plus probiotics group compared to the untreated group, whereas no significant difference was observed in the therapeutic agents plus PMC94 group. These differences in microbial community structure were reflected in the PCoA plots based on (**C**) Bray–Curtis and (**D**) Jensen–Shannon distances. Distinct clustering was observed among the untreated, therapeutic agents-treated, and therapeutic agents plus probiotics groups, whereas the therapeutic agents plus PMC94 group overlapped with the untreated group. Alpha diversity indices, including (**E**) Fisher’s alpha and (**F**) Simpson index, showed significant differences (* *p* < 0.05) between the untreated group and both the therapeutic agents-treated and therapeutic agents plus probiotics groups. In contrast, no significant difference was observed in the therapeutic agents plus PMC94 group.

The ex vivo HGMC demonstrated a clear treatment-dependent modulation of gut microbial diversity and community structure (Figure 3). This integrated platform enables simulation of human gut conditions by combining continuous microbial fermentation with co-culture assays, allowing simultaneous assessment of microbiota dynamics, therapeutic agent responses, and probiotic-mediated interactions under controlled experimental conditions (Figure 3A). Beta diversity analysis showed significant compositional differences between the untreated control and the microbiota treated with therapeutic agents, as indicated by Bray–Curtis (*p* = 0.027) and Jensen–Shannon (*p* = 0.005) distances (Figure 3B–D). While the therapeutic agents plus probiotics group also remained significantly different from the untreated control, the therapeutic agents plus PMC94 group showed no significant difference (Bray–Curtis *p* = 0.249; Jensen–Shannon *p* = 0.121), indicating recovery toward a control-like microbial structure. Consistently, alpha-diversity analysis demonstrated that drug exposure altered microbial richness and diversity, whereas supplementation with PMC94 restored microbial balance (Figure 3E,F). The Fisher and Simpson indices analysis demonstrated that therapeutic agent treatment significantly disrupted microbial diversity (* *p* < 0.05). Co-administration of therapeutic agents with probiotics partially shifted the microbial community toward eubiosis; however, the community structure remained significantly different from the eubiotic state (* *p* < 0.05). Notably, therapeutic agents plus PMC94 showed diversity patterns closer to those of the untreated control condition, suggesting restoration and stabilization of microbial community structure.

### 3.4. Improvement of Gut Microbial Composition Altered by Therapeutic Agents by P. vulgatus PMC94

**Figure 4:**
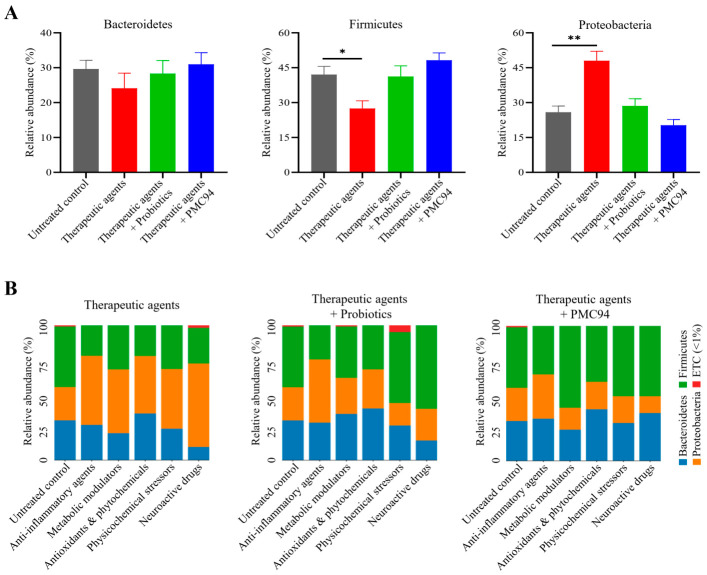
**Taxonomic composition of gut microbial communities in response to therapeutic agents.** Taxonomic changes in the gut microbial community were analyzed in an ex vivo HGMC system following treatment with therapeutic agents, administered alone or in combination with conventional probiotics or post-NGP. (**A**) At the phylum level, treatment with therapeutic agents significantly decreased *Firmicutes* and increased *Proteobacteria*. These alterations were restored upon co-administration with conventional probiotics, and a similar but more pronounced restoration was observed with PMC94. *Bacteroidetes* did not show significant differences among groups but did exhibit trends consistent with those of other phyla. (**B**) Taxonomic profiles for the five representative classes of therapeutic agents—anti-inflammatory agents, metabolic modulators, antioxidants and phytochemicals, physicochemical stressors, and neuroactive drugs—were presented. Although the magnitude of changes varied, similar trends were observed across treatments. Statistical significance among groups (* *p* < 0.05, ** *p* < 0.01) was determined using the Wilcoxon rank-sum test.

Taxonomic composition across untreated control, therapeutic agents alone, therapeutic agents combined with probiotics, and therapeutic agents combined with PMC94 revealed distinct differences in microbial community modulation at the phylum level (Figure 4). Therapeutic agent treatment altered gut microbial composition by significantly decreasing *Firmicutes* (* *p* < 0.05), and markedly increasing *Proteobacteria* (** *p* < 0.01) compared with the untreated control (Figure 4A). Probiotics partially restored microbial balance, whereas PMC94 showed a stronger effect, increasing *Firmicutes* and reducing *Proteobacteria* toward a eubiotic profile comparable to that of the control. Among individual therapeutic agent classes, the untreated control group showed a balanced microbial structure, dominated by *Firmicutes* (44.7%), followed by *Bacteroidetes* (29.7%) and *Proteobacteria* (24.4%) (Figure 4B). Treatment with therapeutic agents alone consistently disrupted microbial balance, leading to a marked increase in *Proteobacteria* (42.3–61.8%) and corresponding decreases in *Firmicutes* (22.8–32.9%) and *Bacteroidetes* (9.7–34.7%). Neuroactive drugs exhibited the strongest dysbiotic effect, with *Proteobacteria* increasing to 61.8% and *Bacteroidetes* decreasing to 9.7%. Similar dysbiotic shifts were observed with anti-inflammatory agents and metabolic modulators, indicating that therapeutic agents broadly promote *Proteobacteria* expansion. Co-administration with conventional probiotics partially restored microbial balance, compared with therapeutic agents alone. In several groups, probiotics reduced *Proteobacteria* abundance (16.5–46.6%) and increased *Firmicutes* (32.8–61.8%), suggesting partial recovery of microbial homeostasis. In contrast, co-administration with PMC94 demonstrated more consistent normalization of microbial composition across therapeutic agent classes. PMC94 markedly reduced *Proteobacteria* (12.3–32.5%) while restoring *Firmicutes* (36.0–60.6%) and *Bacteroidetes* (23.3–38.4%) to levels comparable to those of the control group. For instance, neuroactive drugs combined with PMC94 reduced *Proteobacteria* from 61.8% to 12.3% while recovering *Bacteroidetes* to 35.6%. Similarly, metabolic modulators combined with PMC94 increased *Firmicutes* to 60.6% and reduced *Proteobacteria* to 16.1%, demonstrating strong recovery of microbial composition. Collectively, therapeutic agents induced pronounced dysbiosis, characterized by increased *Proteobacteria* and reduced *Firmicutes*, whereas PMC94 showed a stronger and more consistent capacity to restore microbial balance across different therapeutic classes.

### 3.5. Microbial Community Structure Analysis Following P. vulgatus PMC94 Treatment in HGMS

**Figure 5:**
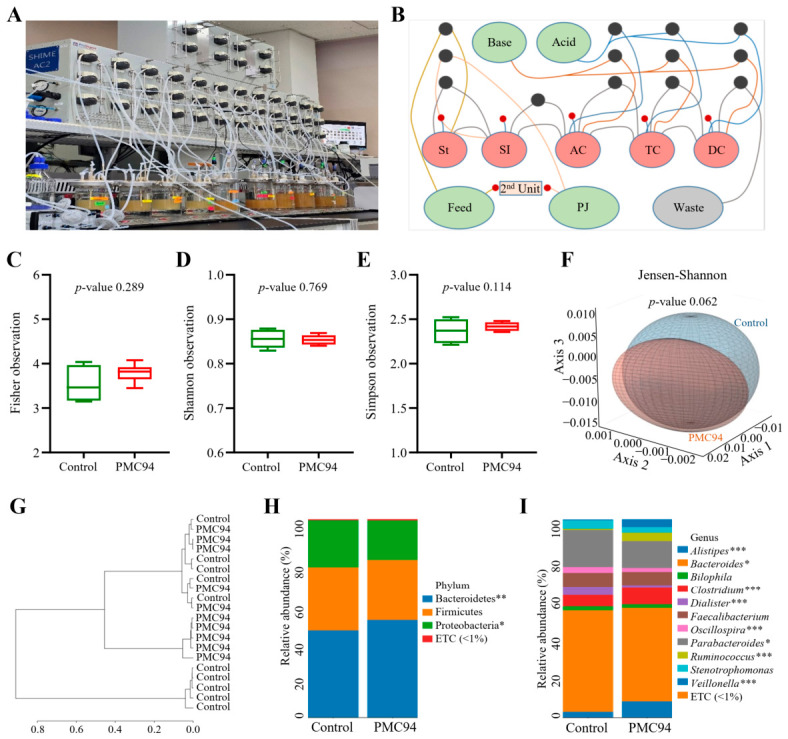
**HGMS-based gut microbiota community profiling following PMC94 treatment.** The experiment was conducted using a twin-unit HGMS system, with one unit serving as the control and the other receiving PMC94 treatment. (**A**) The HGMS setup and (**B**) a schematic illustration of the HGMS system show the sequential passage through the stomach (St), small intestine (SI), and three colon compartments: ascending colon (AC), transverse colon (TC), and descending colon (DC). Alpha diversity metrics, including (**C**) Fisher’s alpha, (**D**) Shannon index, and (**E**) Simpson index, showed no significant differences between the control and PMC94 groups. (**F**) Likewise, beta-diversity analysis based on the Jensen–Shannon distance revealed substantial overlap in overall microbial community structure, indicating no significant separation between the groups. (**G**) UPGMA clustering further demonstrated that the overall microbial community compositions were comparable between the two groups. (**H**,**I**) Further taxonomic analysis revealed several significant shifts in major bacterial groups. Notably, PMC94 increased *Bacteroides* while decreasing *Proteobacteria*. Statistical significance was assessed using the Wilcoxon rank-sum test (* *p* < 0.05, ** *p* < 0.01, and *** *p* < 0.001).

The effects of PMC94 on gut microbial community structure were assessed using a twin-unit HGMS system (Figure 5). The experimental design included parallel control and PMC94-treated systems, both operated under the same conditions (Figure 5A). A schematic overview of the HGMS configuration depicts the dynamic flow of nutrients, treatments, and microbial content through relevant compartments, including the stomach, small intestine, and three sequential colon regions (ascending, transverse, and descending) (Figure 5B). Considering alpha diversity, species richness was measured by Fisher’s alpha, which showed no statistically significant differences between the control and PMC94-treated groups (*p* = 0.289) (Figure 5C). However, there was a slight trend indicating increased diversification in the PMC94-treated group. Similarly, community diversity was assessed with the Shannon and Simpson indices, which remained comparable between the two groups (*p* values of 0.769 and 0.114, respectively) (Figure 5D,E). Moreover, principal coordinate analysis (PCoA) based on Jensen–Shannon divergence revealed partial overlap between control and PMC94-treated samples, indicating modest compositional shifts (Figure 5F). Hierarchical clustering using UPGMA did not reveal a distinct clustering pattern between the control and PMC94-treated groups, with comparable microbial community composition (Figure 5G). Taxonomic profiling showed that PMC94 significantly increased *Bacteroidetes* abundance (43.7% vs. 49.0%; ** *p* < 0.01) and decreased dysbiosis-associated *Proteobacteria* (23.9% vs. 20.0%; * *p* < 0.05), while *Firmicutes* remained relatively unchanged, suggesting selective phylum-level modulation (Figure 5H and Table S3). At the genus level, PMC94 significantly enriched taxa including *Alistipes*, *Clostridium*, *Ruminococcus*, and *Veillonella* (*** *p* < 0.001) (Figure 5I). In contrast, genera such as *Parabacteroides* (* *p* < 0.05) and *Dialister* (*** *p* < 0.001) were significantly reduced following PMC94 treatment. Collectively, PMC94 exhibits a microbiome-safe profile while showing taxonomic changes consistent with those observed in the ex vivo HGMC.

### 3.6. P. vulgatus PMC94-Mediated Microbial Remodeling and SCFA Profiles

**Figure 6:**
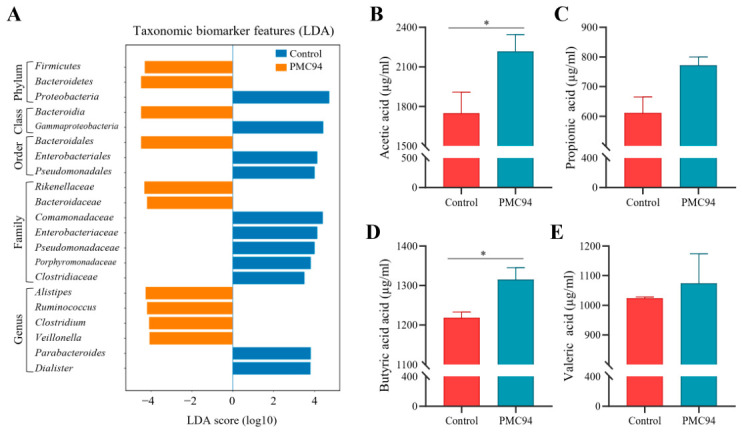
**PMC94-associated taxonomic biomarkers and short-chain fatty acid (SCFA) production.** (**A**) The LEfSe analysis reveals taxonomic cladograms that highlight significant biomarker groups, with a Kruskal–Wallis *p*-value < 0.05. *Firmicutes*, *Bacteroidetes*, and their related taxa were significantly enriched following PMC94 treatment, indicating a shift toward a beneficial microbial profile associated with SCFAs. SCFA production in the PMC94-treated unit, including (**B**) acetate, (**C**) propionate, (**D**) butyrate, and (**E**) valerate, was measured on the end day (day 7). Although an overall increase was observed for all detected SCFAs across treatment groups, acetate and butyrate showed a statistically significant increase. Statistical significance (* *p* < 0.05) was evaluated by an unpaired t-test (GraphPad Prism 9.1.1).

Selective changes in taxonomic biomarkers associated with short-chain fatty acid (SCFA) production were induced by PMC94 in HGMS (Figure 6). According to LEfSe analysis, at the phylum level, *Firmicutes* and *Bacteroidetes* were identified as increased biomarkers, whereas *Proteobacteria* was identified as a decreased biomarker following PMC94 treatment (Figure 6A). At the genus level, *Alistipes*, *Ruminococcus*, *Clostridium*, and *Veillonella* were identified as increased biomarkers, while *Parabacteroides* and *Dialister* were identified as decreased biomarkers. Notably, the major biomarkers increased by PMC94 correspond to SCFA-producing bacteria; therefore, SCFA levels were further measured. An endpoint comparison at day 7 confirmed enhanced SCFA production following PMC94 treatment. Compared to the control, the PMC94-treated unit exhibited higher concentrations of acetate (2217 vs. 1748 µg/mL) (Figure 6B), propionate (772 vs. 611 µg/mL) (Figure 6C), and butyrate (1315 vs. 1218 µg/mL) (Figure 6D). Valerate levels were also elevated in the PMC94 group (1074 µg/mL) compared to controls (1024 µg/mL), although this increase did not achieve statistical significance at the endpoint (Figure 6E). The quantitative results indicate that PMC94 treatment enhances microbial SCFA production, particularly affecting butyrate and acetate levels. Notably, butyrate and acetate displayed a significant response to PMC94 treatment, showing a clear increase at day 7 (* *p* < 0.05).

### 3.7. Targeted Microbiota Modulation by P. vulgatus PMC94 and Gut Function in Mice

**Figure 7:**
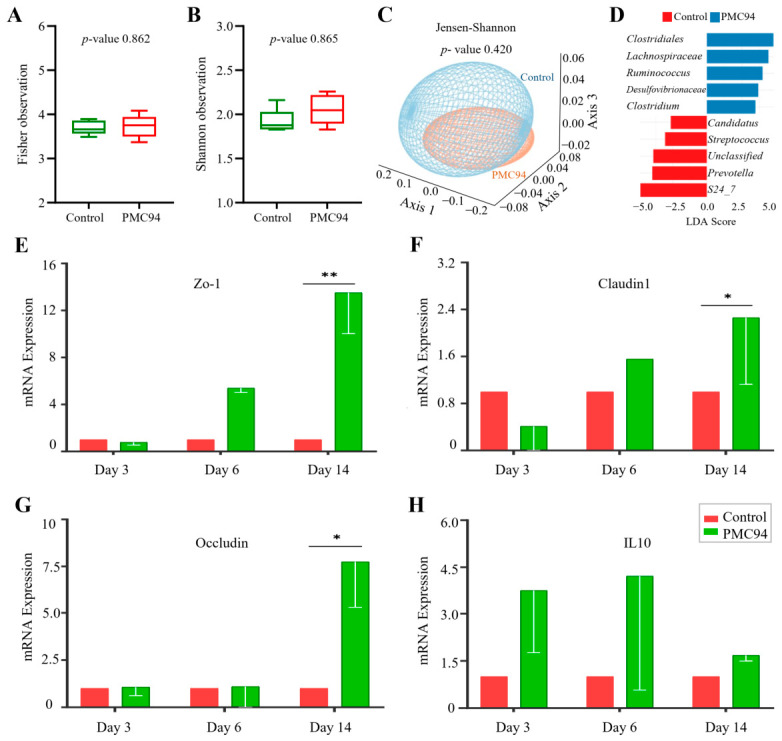
**PMC94-mediated modulation of the gut microbiota and intestinal function in mice.** PMC94-induced microbial community profiles were compared with the control group. Alpha diversity indices, including (**A**) Fisher’s alpha and (**B**) Shannon, show no significant changes in richness or diversity. (**C**) Similarly, beta-diversity analysis based on Jensen–Shannon divergence reveals substantial overlap in overall microbial structure. (**D**) LEfSe analysis identifies enrichment of SCFA-associated taxa, including *Clostridiales*, *Lachnospiraceae*, and *Ruminococcus* in the PMC94 group. Considering gut function, relative mRNA expression levels of tight junction-associated genes, (**E**) ZO-1, (**F**) Claudin-1, and (**G**) Occludin, exhibited similar trends, with remarkably increased expression following PMC94 treatment. (**H**) The anti-inflammatory cytokine IL-10 in gut tissues also showed an overall increase over time following PMC94 treatment. Statistical significance (* *p* < 0.05, ** *p*< 0.01) was evaluated with the unpaired t-test (GraphPad Prism 9.1.1).

The in vivo effects of PMC94 on gut microbiota were assessed using a murine model (Figure 7). Considering the overall community structure, alpha diversity metrics reflecting microbial richness and diversity showed no significant differences between the groups (Fisher (*p* = 0.862) and Shannon (*p* = 0.865)) (Figure 7A,B). Consistently, beta diversity analysis further demonstrated substantial overlap in global microbial structure between control and PMC94-treated samples, confirming the maintenance of an overall microbial community structure (Figure 7C). Despite the comparable overall diversity, LDA analysis revealed selective taxonomic modulation following PMC94 treatment (Figure 7D). PMC94 was associated with enrichment of beneficial taxa, including members of *Clostridiales*, *Lachnospiraceae*, and *Ruminococcus*, supporting the previous trend. Functional validation using host-response markers demonstrated a significant enhancement in gut barrier-associated gene expression following PMC94 treatment. Treatment resulted in a time-dependent upregulation of tight junction–related genes. Notably, the expression of ZO-1 significantly increased in the PMC94 group, becoming evident by day 6 and reaching a marked elevation by day 14 compared to the control group (** *p* < 0.01) (Figure 7E). A similar pattern was observed for Claudin-1, with PMC94-treated mice showing significantly higher expression at day 14 relative to controls (* *p* < 0.05) (Figure 7F). Additionally, Occludin expression increased following PMC94 administration, exhibiting a significant rise by day 14 compared to the control group (* *p* < 0.05) (Figure 7G). Furthermore, PMC94 also modulated IL-10 expression (Figure 7H). IL-10 expression was elevated in the PMC94-treated group at days 3 and 6 compared to controls. Although IL-10 expression declined from the peak observed at day 6 to day 14, it remained higher than that of the control group, underscoring the sustained immunomodulatory effects.

### 3.8. Safety Evaluation of P. vulgatus PMC94 in BALB/c Mice

**Figure 8:**
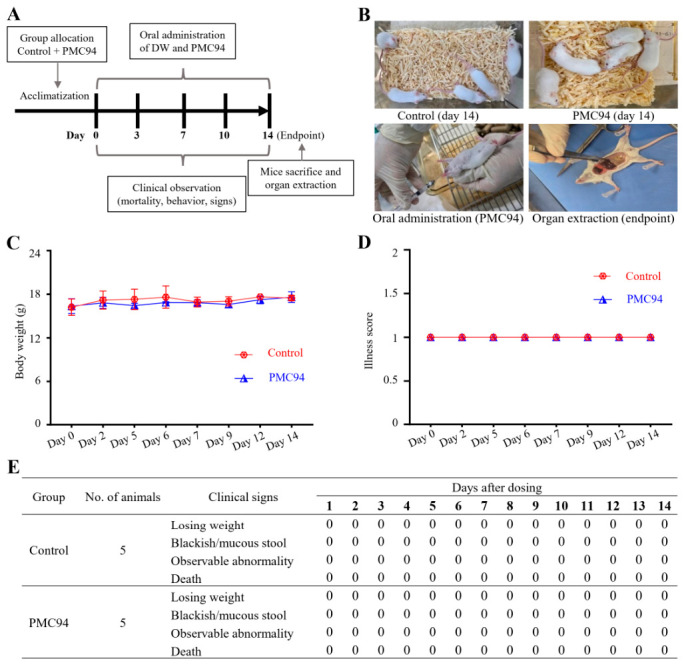
**Repeated oral toxicity assessment of PMC94 in mice.** (**A**,**B**) BALB/c mice were divided into control and PMC94-treated groups and orally administered saline or PMC94 once daily for 14 days, followed by clinical observation throughout the study and organ collection at the endpoint; representative images of the experimental process, including animal condition, oral administration, and organ extraction, are shown. Six-week-old BALB/c mice were used, with five animals per group (n = 5). (**C**) Body weight changes and (**D**) illness scores showed no significant differences between the two groups during the experimental period. (**E**) No toxic clinical signs, including weight loss, blackish or mucous stool, observable abnormalities, or death, were observed in either group throughout the study period.

The in vivo safety of PMC94 was assessed in a 14-day oral administration study in BALB/c mice (Figure 8). After acclimatization, the mice were divided into control and PMC94-treated groups (Figure 8A,B). Their general health, clinical signs, and changes in body weight were monitored throughout the experiment. Body weight was measured at multiple time points during the study. Both control and PMC94-treated mice showed a consistent and similar increase in body weight over the 14 days, with no statistically significant differences between the groups at any time point (Figure 8C). Additionally, illness scores were recorded to evaluate any potential treatment-related clinical symptoms (Figure 8D). Throughout the study, illness scores remained low and comparable between the control and PMC94-treated groups. No signs of weight loss, abnormal stool characteristics (e.g., blackish or mucous-containing stool), behavioral abnormalities, or mortality occurred in either group, further supporting the in vivo safety of PMC94 under the tested conditions (Figure 8E).

## 4. Discussion

Probiotics based on lactic acid bacteria, derived from traditional fermented foods humans have consumed for centuries, have been widely applied to prevent and treat various diseases, and, more recently, to therapeutic development [15,47]. However, this approach has largely relied on a limited set of cultivable strains [47]. As its application has expanded beyond gastrointestinal disorders to a broader spectrum of systemic diseases, the limitations of conventional lactic acid bacteria-based strategies have become increasingly evident [48]. Specifically, constraints in strain diversity and functional capacity limit their ability to adequately recapitulate the complexity and functional demands of the gut microbial ecosystem [48,49]. To address these limitations, attention has shifted toward next-generation probiotics (NGP), including *Akkermansia* and *Faecalibacterium*, which have been more recently characterized and are relatively difficult to culture [8]. Nevertheless, current NGP approaches remain focused on a limited set of taxa and are insufficient to fully capture the ecological complexity and functional interactions inherent to the gut microbiome [50]. Accordingly, a conceptual transition beyond strain-centric approaches toward a functionally and ecologically informed framework, herein referred to as Post-NGP, is required.

In this study, we propose *P. vulgatus* as a representative Post-NGP candidate. *P. vulgatus*, an obligate anaerobe widely distributed in the healthy human gut, is a key functional member of the gut microbial ecosystem with substantial potential for microbiome-based therapeutic applications [51]. Previous studies have shown that *P. vulgatus* plays an important role in complex carbohydrate metabolism and short-chain fatty acid production, thereby contributing to the gut’s metabolic landscape, and has also been associated with modulation of host immune responses [51,52,53]. However, these functional roles have been reported to vary with host conditions and microbial community context, suggesting strain- and environment-dependent characteristics. Here, PMC94 was successfully isolated from human fecal samples using a strict anaerobic cultivation strategy within a culture-based discovery framework, reflecting a culturomics-oriented approach to capture gut microbial diversity that is often underrepresented in conventional analyses. Notably, despite its prevalence as a commensal species, most previous studies on *P. vulgatus* have relied on metagenomic association analyses, with relatively limited efforts to isolate and functionally validate individual strains, thereby constraining its translational application [54]. The isolate exhibited stable and reproducible growth characteristics, supporting its suitability for downstream functional evaluation. Furthermore, whole-genome sequencing confirmed its taxonomic classification within *P. vulgatus* and revealed genomic features distinct from those of previously reported strains. Comparative genomic analysis further demonstrated differences in genome size, coding sequence composition, and gene content relative to reference strains, supporting its classification as a genetically distinct strain within the species. Taken together, these findings suggest that the present approach, integrating targeted anaerobic cultivation and genome-based characterization, is consistent with the Post-NGP concept proposed in this study, which emphasizes the systematic identification and validation of microbiome-derived therapeutic candidates based on functional and ecological relevance.

With the advent of the microbiome era, it has become increasingly recognized that a wide range of human diseases are closely associated with gastrointestinal health [2,55]. Although microbiome-based approaches have expanded from functional foods to therapeutic interventions across diverse diseases, their fundamental basis remains rooted in the gut, particularly in relation to immune homeostasis [56]. While research trends are increasingly extending beyond gastrointestinal disorders to extra-intestinal diseases, gut health remains a central determinant of systemic health, underscoring its critical importance [57]. Advances in metagenomic technologies have further reinforced the concept that the balance of the intestinal microbiome largely governs gut health [58]. Whereas earlier studies primarily focused on specific beneficial or pathogenic species, accumulating evidence suggests that the broader microbial community and its composition contribute integratively through complex interspecies interactions and ecosystem-level functions [59,60]. Against this backdrop, increasing attention has been directed toward the impact of therapeutic agents, which are routinely and often indiscriminately used in modern clinical settings, on the gut microbiome. Historically, the efficacy and adverse effects of such agents were interpreted mainly based on their direct interactions with host targets [61]; however, it has recently been demonstrated that gut microbiota can directly influence drug metabolism, bioavailability, and biotransformation into active metabolites, and growing evidence indicates that inter-individual variability in drug efficacy and safety is, at least in part, attributable to differences in gut microbiome composition [62]. Accordingly, understanding the gut microbiome has emerged as a critical factor for predicting therapeutic responses and developing personalized treatment strategies [56]. Despite these advances, most studies have focused on individual drugs or limited drug classes, and comprehensive evaluations of microbiome perturbations across diverse therapeutic categories remain limited. In parallel, probiotics, which have long been consumed as dietary supplements, are now recognized not only for their roles in beneficial metabolite production, immune modulation, and suppression of pathogenic bacteria, but also for their broader contribution to the maintenance and restoration of gut microbiome homeostasis [63]. However, their effectiveness in restoring microbiome disturbances induced by diverse therapeutic agents remains incompletely understood, particularly in comparison with emerging next-generation or anaerobic microbial candidates [6,64]. Within this context, as a Post-NGP candidate, *Phocaeicola vulgatus* can be positioned as a key microbial species capable of modulating the gut microbiome and promoting intestinal health as a primary functional basis. Therefore, in this study, we aimed to evaluate microbiome perturbations induced by representative therapeutic classes systematically and to comparatively assess the potential of restoring conventional probiotics and the anaerobic candidate strain *P. vulgatus*.

The ex vivo HGMC system established in this study was designed as a platform that preserves key features of the gut microbial ecosystem while allowing controlled experimental manipulation, thereby enabling precise evaluation of drug–microbiome interactions. To systematically evaluate the efficacy of microbiome modulation, the ex vivo HGMC was applied in this study. The objective was to determine whether the candidate strain could restore drug-induced gut microbial dysbiosis and to compare its effects with those of conventional probiotics directly. While previous studies have primarily focused on antibiotic-induced dysbiosis or the effects of individual drugs [65,66], we evaluated microbiome alterations across multiple therapeutic classes, including anti-inflammatory agents, metabolic modulators, antioxidants and phytochemicals, physicochemical stressors, and neuroactive drugs. These diverse therapeutic agents commonly induced dysbiosis, characterized by reduced microbial diversity and altered community structure, consistent with previous reports indicating that non-antibiotic drugs can also perturb the gut microbiome [65,66,67]. Thus, our results extend the concept of drug-induced dysbiosis beyond antibiotics to a broader range of therapeutic agents. Co-administration of probiotics partially restored microbial diversity and shifted the community toward a more balanced state, consistent with previous studies demonstrating probiotics’ modulatory effects on gut microbiota composition [68,69]. In contrast, PMC94 treatment resulted in the greatest improvement in both alpha and beta diversity indices, indicating superior restoration of microbial structure. Importantly, this study demonstrates that drug-induced dysbiosis across multiple therapeutic classes can be comparatively evaluated within a single, experimentally controlled HGMC platform established in this work, and that recovery responses can be systematically assessed within the same framework, underscoring the significance of this approach. Given the critical role of microbiome balance, or eubiosis, in host health and disease, these findings highlight the potential importance of targeted microbiome restoration strategies.

Therapeutic agents induced a decrease in *Firmicutes* and an increase in *Proteobacteria*, a pattern interpreted as a typical dysbiotic state reflecting disruption of gut microbial homeostasis [70,71]. In particular, the increase in *Proteobacteria* suggests an unstable gut environment and selective ecological disturbance [71]. In contrast, the reduction in *Firmicutes* may reflect impaired fermentative metabolism and loss of functions associated with maintaining microbial homeostasis [72]. A decreasing trend was also observed for *Bacteroidetes*, which play a key role in complex carbohydrate degradation and metabolic balance, indicating a broader weakening of the functional foundation of the gut microbiome [73]. These alterations are not confined to specific taxa but rather reflect a global imbalance of the microbial community, and thus warrant further investigation at finer taxonomic resolution. Furthermore, these structural changes were consistently observed across all therapeutic classes, although the magnitude and specific patterns varied depending on the type of agents. Anti-inflammatory agents showed a relatively pronounced increase in *Proteobacteria* and a decrease in *Firmicutes*, suggesting that even inflammation-modulating drugs can directly perturb the gut microbial ecosystem [74]. Metabolic modulators appeared to induce compositional shifts associated with bile acid and lipid metabolism, while antioxidants and phytochemicals also selectively altered microbial structure, likely through changes in the redox environment rather than purely protective effects [75,76]. Physicochemical stressors likely drove broader community restructuring by altering the gut microenvironment itself, and neuroactive drugs may have influenced microbial composition through indirect mechanisms involving the gut–brain axis [77]. Taken together, these findings extend the concept of drug-induced dysbiosis beyond antibiotics, demonstrating that diverse classes of therapeutic agents can commonly destabilize gut microbial structure. Notably, differences in recovery patterns were also observed. Co-administration of conventional probiotics partially restored the decrease in *Firmicutes* and the increase in *Proteobacteria*; however, the extent of recovery varied across therapeutic classes and remained limited in some cases. In contrast, PMC94 more effectively suppressed *Proteobacteria* and consistently restored *Firmicutes* across most conditions, suggesting a more robust capacity to re-establish microbial balance [71,72]. Therefore, these results indicate that PMC94 may provide enhanced structural resilience against heterogeneous dysbiosis induced by diverse therapeutic agents.

To further interpret the findings from the ex vivo HGMC system, we employed the HGMS platform to evaluate the direct effects of PMC94 on a stable gut microbiota under baseline conditions. Notably, PMC94 did not induce significant changes in alpha or beta diversity, indicating that it does not disrupt overall microbial community structure and thus exhibits a microbiome-safe profile. Despite this global stability, significant shifts were observed at the taxonomic level. Within the *Firmicutes* phylum, genera such as *Ruminococcus* and *Oscillospira* were significantly increased, suggesting selective enrichment of taxa associated with short-chain fatty acid production and anti-inflammatory functions [78,79]. In the *Bacteroidetes* phylum, significant changes were observed in *Bacteroides* and *Parabacteroides*, with the increase in *Bacteroides* likely reflecting enhanced carbohydrate metabolism and maintenance of metabolic homeostasis [80]. In contrast, the opportunistic genus *Stenotrophomonas*, associated with *Proteobacteria*, showed a decreasing trend, indicating suppression of dysbiosis-associated taxa and stabilization of the gut environment [81]. Additionally, significant changes were observed in genera such as *Alistipes*, *Dialister*, and *Veillonella*, which are known to be sensitive to shifts in host and microbial conditions, suggesting that PMC94 selectively modulates ecologically responsive taxa. In this context, these findings indicate that PMC94 does not broadly restructure the gut microbiome but instead modulates functionally relevant taxa. Importantly, many of the genera altered in the HGMS system overlap with those implicated in dysbiosis and recovery in the HGMC model, supporting the notion that the restorative effects of PMC94 are mediated through selective enrichment of beneficial microbial groups and suppression of opportunistic taxa.

Biomarker analysis revealed a distinct microbial signature in the PMC94-treated group, characterized by the selective enrichment and reduction in specific taxa, reflecting a functional shift in the gut microbiome rather than simple compositional changes. Notably, genera within the *Firmicutes* phylum, including *Ruminococcus* and *Clostridium*, which are well-established butyrate producers, were enriched, while *Veillonella*, also belonging to *Firmicutes*, is known to participate in propionate production via lactate utilization, representing a key metabolic link within SCFA-producing networks [78,79]. In parallel, taxa within the *Bacteroidetes* phylum, such as *Alistipes* and *Bacteroides*-related genera, which are associated with complex carbohydrate degradation and acetate production, further support the enrichment of fermentative metabolic capacity [80,82]. In contrast, the reduction in genera such as *Parabacteroides* and *Dialister*, both of which exhibit context-dependent roles in gut ecology, may reflect selective suppression of taxa associated with dysbiotic or competing metabolic states [83]. Collectively, this pattern of enrichment and depletion represents a coordinated restructuring of microbial interactions and metabolic networks favoring SCFA production. This microbial signature was consistent with the metabolomic profile, which showed significant increases in acetate and butyrate. Acetate serves as a central metabolite in microbial cross-feeding networks, providing substrates for downstream fermentation processes [84]. In contrast, butyrate is a primary energy source for colonocytes and plays a crucial role in maintaining epithelial barrier integrity and regulating inflammatory responses [85]. Importantly, SCFAs are not merely metabolic outputs but can function as active modulators of the gut ecosystem [86]. Through engagement of G-protein-coupled receptors (e.g., GPR41 and GPR43), SCFAs contribute to immune regulation and the establishment of an anti-inflammatory milieu, while also lowering luminal pH to suppress the expansion of opportunistic taxa such as *Proteobacteria* and promoting a stable, SCFA-producing microbial community [87]. Therefore, the observed increase in SCFAs should not be interpreted solely as a downstream consequence of microbial changes, but rather as a functional axis that actively contributes to the transition of the gut microbiome from a dysbiotic to an eubiotic state [49]. In the context of the ex vivo HGMC findings, where PMC94 effectively restored drug-induced dysbiosis, the enrichment of SCFA-associated taxa and the concomitant increase in acetate and butyrate provide mechanistic support for its restorative capacity. Taken together, PMC94 appears to promote a microbiome configuration that enhances SCFA-centered metabolic interactions, thereby facilitating the stabilization of the gut ecosystem and the recovery of microbial homeostasis.

To extend the findings from the ex vivo HGMC and HGMS systems into a physiological context, we further evaluated the effects of PMC94 in vivo using a murine model. This in vivo analysis was designed to determine whether microbiome-level changes translate into host functional outcomes. Notably, PMC94 treatment did not induce significant alterations in either alpha or beta diversity, indicating preservation of overall microbial community structure and confirming an in vivo microbiome-safe profile, consistent with observations from the HGMS system. Despite this global stability, LEfSe-based biomarker analysis revealed a distinct microbial signature characterized by the selective enrichment of SCFA-associated taxa, including *Clostridiales*, *Lachnospiraceae*, and *Ruminococcus* [88]. These taxa, predominantly affiliated with the *Firmicutes* phylum, are well recognized for their roles in butyrate production and can therefore be interpreted as functional biomarkers reflecting enhanced SCFA-related metabolic capacity. Importantly, these microbial changes were accompanied by significant upregulation of tight junction-associated genes, including ZO-1, Claudin-1, and Occludin, as well as increased expression of the anti-inflammatory cytokine IL-10. Given that SCFAs, particularly butyrate, are known to regulate epithelial barrier integrity and immune homeostasis, these findings support a functional link between PMC94-induced microbial modulation and improved host barrier function and immune regulation [89,90]. Although differences in gut microbiota composition between mice and humans should be considered, the present results suggest that PMC94 promotes a functionally favorable microbiome configuration characterized by SCFA-associated taxa. This microbiome state is associated with enhanced barrier integrity and anti-inflammatory responses, thereby supporting the transition from dysbiosis toward eubiosis [91].

To establish the translational feasibility of PMC94 as a Post-NGP candidate, its safety profile must be considered thoroughly [92]. The in vivo safety of PMC94, following oral administration in BALB/c mice, was evaluated during continuous treatment. PMC94-treated mice displayed normal behavior, steady body weight gain comparable to controls, and consistently low illness scores, indicating no treatment-related toxicity or distress. Importantly, no cases of mortality or adverse clinical signs were observed, supporting the good tolerability of PMC94 under repeated dosing conditions [18]. These findings are particularly significant given that PMC94 is an obligate anaerobe derived from the human gut, as safety remains a critical bottleneck in NGP development [93]. The absence of negative physiological or behavioral outcomes confirms that PMC94-mediated modulation of the microbiota and host benefits does not result in systemic or gastrointestinal adverse effects. This outcome demonstrates that PMC94 is safe for oral administration in vivo, fulfilling a key prerequisite for further preclinical development and potential clinical translation [93].

Although this study provides comprehensive evidence supporting the probiotic potential of *P. vulgatus* PMC94, several limitations should be acknowledged. Genome characterization and microbiome profiling were performed using earlier-generation sequencing platforms, and future studies may benefit from newer high-throughput sequencing technologies with greater throughput and resolution. In addition, further studies incorporating physiologically relevant conditions, more comprehensive analytical approaches, and direct in vivo comparisons with therapeutic agents are warranted to further validate the therapeutic potential of PMC94. Despite these limitations, this study demonstrates that *P. vulgatus* PMC94 is a distinct, safe, and robust Post-NGP candidate. It effectively restores gut microbiota balance by selectively suppressing dysbiosis-associated *Proteobacteria* and enriching beneficial anaerobic commensals. By maintaining overall microbial stability while addressing compositional and metabolic imbalances, PMC94 enhances short-chain fatty acid production, strengthens intestinal barrier integrity, and exhibits immunomodulatory effects in vivo. These findings position PMC94 as a promising precision microbiome stabilizer for microbiome-based therapeutic development.

## 5. Conclusions

**Figure 9:**
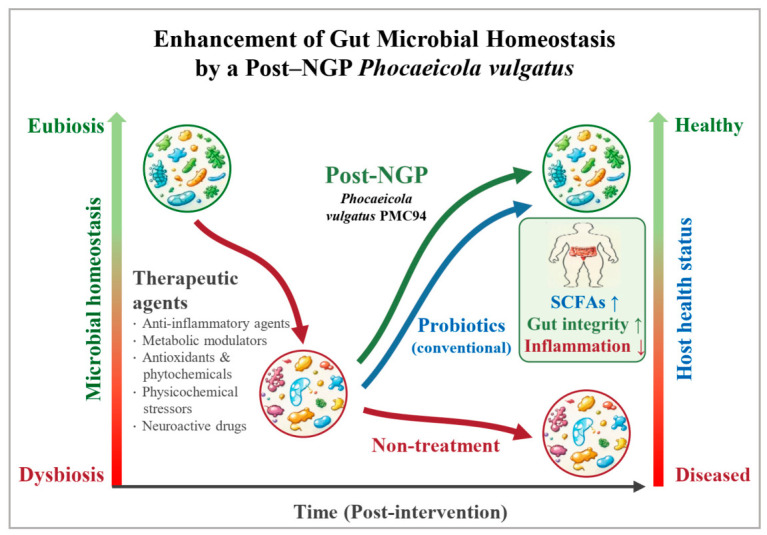
**Schematic overview of gut microbiome homeostasis improvement by Post-NGP *Phocaeicola vulgatus*.** Various therapeutic agents, including anti-inflammatory agents, metabolic modulators, antioxidant and phytochemical compounds, chemical or physicochemical stressors, and neuroactive drugs, disrupt gut microbial balance, leading to dysbiosis characterized by reduced beneficial bacteria and increased opportunistic pathogens. Co-administration of conventional probiotics partially alleviates this imbalance and promotes recovery of the gut microbiota. Notably, the Post-NGP candidate strain PMC94 demonstrates a greater capacity to restore the microbiome toward an eubiotic state than conventional probiotics. These findings suggest that PMC94 has the potential to mitigate therapy-induced dysbiosis and to contribute to the restoration of host health.

This study identifies *P. vulgatus* PMC94 as a functionally validated Post-NGP candidate capable of restoring drug-induced dysbiosis through targeted modulation of the gut microbiome without disrupting overall community stability. This effect was characterized by the selective enrichment of SCFA-associated microbial taxa and the increased production of key SCFAs, particularly acetate and butyrate, which were functionally linked to enhanced intestinal barrier integrity and immune regulation. In addition, PMC94 exhibited a microbiome-safe profile, maintaining microbial diversity without inducing detectable toxicity or adverse effects. Collectively, these findings suggest that PMC94 promotes the transition from dysbiosis to eubiosis by reprogramming SCFA-centered microbiome–host interactions, thereby providing a mechanistically grounded strategy for microbiome-based therapeutic development. The overall workflow and key concepts of this study are summarized in Figure 9.

## Figures and Tables

**Table 1:** 16s rRNA gene sequencing and BLAST analysis results in relation to corresponding NCBI records.

NCBI Reference	Organism	Length	Score	Identities	Gaps
NR_074515.1	*Phocaeicola vulgatus* ATCC 8482	1510	2632 bits (1425)	1435/1440 (99%)	0/1440 (0%)
NR_112946.1	*Phocaeicola vulgatus* strain JCM 5826	1489	2632 bits (1425)	1435/1440 (99%)	0/1440 (0%)
NR_041351.1	*Phocaeicola dorei* strain 175	1493	2436 bits (1319)	1405/1446 (97%)	8/1446 (1%)
NR_113064.1	*Phocaeicola sartorii* JCM 16497	1490	2200 bits (1191)	1364/1447 (94%)	13/1447 (1%)
NR_113195.1	*Phocaeicola sartorii* JCM 17136	1490	2200 bits (1191)	1364/1447 (94%)	13/1447 (1%)
NR_181010.1	*Phocaeicola faecalis* strain FXJYN30E22	1412	2115 bits (1145)	1325/1412 (94%)	11/1412 (1%)
NR_041278.1	*Phocaeicola coprocola* strain M16	1490	2067 bits (1119)	1333/1440 (93%)	0/1440 (0%)
NR_179635.1	*Bacteroides cutis* strain Marseille-P4118	1485	2054 bits (1112)	1340/1449 (92%)	19/1449 (1%)
NR_113072.1	*Bacteroides rodentium* JCM 16496	1488	2049 bits (1109)	1333/1443 (92%)	7/1443 (0%)

NCBI, National Center for Biotechnology Information.

**Table 2:** Comparison of the chromosomal properties of *Phocaeicola vulgatus* strains.

Strain	PMC94	MG01-03	MG01-10	VIC01	BGF-191	CavFT-hAR107	Bv4543
Sources	Human feces	Homo sapiens	Human feces	Mus musculus	Peritoneal Fluid	Gut-wall	Feces
Genome size (bp)	5,120,799	5,073,285	4,957,189	5,010,342	5,215,841	5,751,554	4,617,721
G+C content (%)	42.2	42.5	42.5	42.5	42.5	42.5	42.5
Predicted CDS	4269	4041	4055	3921	4376	4294	3666
Number of rRNA genes	21	21	21	21	23	22	14
Number of tRNA genes	81	81	87	80	86	91	67

MG01-03: https://www.ncbi.nlm.nih.gov/nuccore/NZ_AP025232.1 (accessed on 18 February 2026); MG01-10: https://www.ncbi.nlm.nih.gov/nuccore/NZ_AP025240.1 (accessed on 18 February 2026); VIC01: https://www.ncbi.nlm.nih.gov/nuccore/NZ_CP043529.1 (accessed on 18 February 2026); BGF-191: https://www.ncbi.nlm.nih.gov/nuccore/NZ_CP081912.1 (accessed on 18 February 2026); CavFT-hAR107: https://www.ncbi.nlm.nih.gov/nuccore/NZ_JAWDHD000000000.1 (accessed on 18 February 2026); Bv4543: https://www.ncbi.nlm.nih.gov/nuccore/NZ_JAKKXC000000000.1 (accessed on 18 February 2026).

## Data Availability

Data contained in the article and the original data supporting the present study’s findings are available from the corresponding author upon reasonable request.

## Supplementary Materials

The following supporting information can be downloaded at: https://www.mdpi.com/article/10.3390/nu18142355/s1, Table S1: 16s rRNA genome sequence of the conventional probiotics and BLAST analysis findings. Table S2: Primer sequences for targeted genes [94,95,96]. Table S3: Taxonomic profile of the samples in the human gut microbiome simulator system.
