# Supplementary material for: Enhancement of Gut Microbial Homeostasis by a Post-NGP Phocaeicola vulgatus
Source: Nutrients. 2026 Jul 17;18(14):2355. doi: 10.3390/nu18142355 (PMC13415010; doi:10.3390/nu18142355)
Supplement: Supplementary file 1 [file nutrients-18-02355-s001.zip › nutrients-4395152-supplementary.pdf]

Supplementary Information

Table S1. 16s rRNA genome sequence of the conventional probiotics and BLAST analysis findings.

Organism	NCBI Reference	Strains	Length	Identities	Gaps
Latilactobacillus sakei DSM	NR_042443.1	Latilactobacillus sakei strain DSM 20017	1561	1494/1496 (99%)	0/1496 (0%)
	NR_113821.1	Latilactobacillus sakei strain NBRC 15893	1499	1489/1490 (99%)	0/1490 (0%)
	NR_042438.1	Latilactobacillus graminis strain G90 (1)	1548	1483/1497 (99%)	2/1497 (0%)
	NR_042437.1	Latilactobacillus curvatus strain DSM 20019	1559	1483/1497 (99%)	5/1497 (0%)
Latilactobacillus curvatus	NR_042437.1	Latilactobacillus curvatus strain DSM 20019	1559	1482/1485 (99%)	1/1485 (0%)
	NR_113334.1	Latilactobacillus curvatus strain NBRC 15884	1491	1477/1479 (99%)	2/1479 (0%)
	NR_042438.1	Latilactobacillus graminis strain G90	1548	1482/1488 (99%)	2/1488 (0%)
	NR_042443.1	Latilactobacillus sakei DSM 20017	1561	1475/1488 (99%)	4/1488 (0%)
Lactococcus lactis	NR_040955.1	Lactococcus lactis strain NCDO 604	1499	1475/1477 (99%)	1/1477 (0%)
	NR_113960.1	Lactococcus lactis strain NBRC 100933	1472	1470/1472 (99%)	1/1472 (0%)
	NR_040956.1	Lactococcus lactis strain NCDO 2181	1499	1473/1477 (99%)	1/1477 (0%)
	NR_116443.1	Lactococcus cremoris strain L105	1541	1471/1482 (99%)	1/1482 (0%)
Latilactobacillus sakei NBRC	NR_113821.1	Latilactobacillus sakei strain NBRC 15893	1499	1457/1457 (100%)	0/1457 (0%)
	NR_042443.1	Latilactobacillus sakei strain DSM 20017	1561	1459/1460 (99%)	0/1460 (0%)
	NR_042437.1	Latilactobacillus curvatus strain DSM 20019	1559	1449/1461 (99%)	4/1461 (0%)
	NR_042438.1	Latilactobacillus graminis strain G90	1548	1447/1460 (99%)	2/1460 (0%)
Bacillus velezensis	NR_075005.2	Bacillus velezensis strain FZB42	1550	1477/1480 (99%)	0/1480 (0%)
	NR_102783.2	Bacillus subtilis subsp. subtilis strain 168	1550	1474/1480 (99%)	0/1480 (0%)
	NR_151897.1	Bacillus nakamurai strain NRRL B-41091	1508	1474/1480 (99%)	0/1480 (0%)
	NR_117274.1	Bacillus siamensis KCTC 13613	1525	1476/1483 (99%)	2/1483 (0%)
Pediococcus pentosaceus	NR_042058.1	Pediococcus pentosaceus strain DSM 20336	1569	1478/1481 (99%)	2/1481 (0%)
	NR_042401.1	Pediococcus stilesii strain FAIR-E 180	1529	1450/1474 (98%)	0/1474 (0%)
	NR_042057.1	Pediococcus acidilactici DSM 20284	1569	1453/1481 (98%)	2/1481 (0%)
	NR_075029.1	Pediococcus claussenii strain ATCC BAA-344	1567	1441/1483 (97%)	4/1483 (0%)

NCBI, National Center for Biotechnology Information.

Table S2. Primer sequences for targeted genes.

Gene Name	Primer	Primer Sequence	References
Occludin	Forward	ATCAACAAAGGCAACTCT	[1]
	Reverse	GCAGCAGCCATGTACTCT	
Zo 1	Forward	GAGTTTGATAGTGGCGTT	[1]
	Reverse	GTGGGAGGATGCTGTTGT	
Claudin 1	Forward	TACTTTCCTGCTCCTGTC	[1]
	Reverse	AAGGCGTTAATGTCAATC	
IL-10	Forward	GCTCTTACTGACTGGCATGAG	[2]
	Reverse	CGCAGCTCTAGGAGCATGTG	
Gapdh	Forward	CATGGCCTTCCGTGTTCCTAC	[3]
	Reverse	TCAGTGGGCCCTCAGATGC	

Ref-

1. Zhang, B., & Guo, Y. (2009). Supplemental zinc reduced intestinal permeability by enhancing occludin and zonula occludens protein-1 (ZO-1) expression in weaning piglets. British journal of nutrition, 102(5), 687-693.
2. Chen, H., Yan, X., Li, Z., Deng, Z., Gu, J., Zeng, F., ... & Zhang, J. (2025). MyD88 orchestrates fatty acid metabolism in tumor-associated macrophages and non-alcoholic fatty liver disease-related hepatocarcinogenesis. Frontiers in Immunology, 16, 1589255.
3. Song, Y. R., Lee, C. M., Lee, S. H., & Baik, S. H. (2021). Evaluation of probiotic properties of Pediococcus acidilactici M76 producing functional exopolysaccharides and its lactic acid fermentation of black raspberry extract. Microorganisms, 9(7), 1364.

Table S3. Taxonomic profile of the samples in the human gut microbiome simulator system.

Taxonomic rank	Taxa designation	Control							PMC94						
		Avg.	SD	Max	Min	1st Q	2nd Q	3rd Q	Avg.	SD	Max	Min	1st Q	2nd Q	3rd Q
Phylum	Actinobacteria	0.2	0.1	0.4	0.1	0.2	0.2	0.3	0.3	0.1	0.4	0.2	0.2	0.3	0.3
	Bacteroidetes	43.7	2.1	46.9	41.2	41.8	43.7	45.0	49.0	1.9	51.2	46.1	47.3	48.9	50.9
	Firmicutes	31.9	0.9	33.0	29.9	31.6	32.0	32.5	30.4	3.6	34.3	26.1	27.2	30.6	33.8
	Not_Assigned	0.2	0.2	0.5	0.0	0.1	0.2	0.3	0.4	0.3	0.9	0.1	0.2	0.2	0.5
	Proteobacteria	23.9	1.8	26.2	20.6	22.9	23.5	25.4	20.0	5.4	26.4	14.1	14.6	20.0	25.3
Class	Actinobacteria	0.2	0.1	0.4	0.1	0.1	0.2	0.3	0.3	0.1	0.4	0.2	0.2	0.3	0.3
	Bacteroidia	43.7	2.1	46.9	41.2	41.8	43.7	45.0	49.0	1.9	51.2	46.1	47.3	48.9	50.9
	Betaproteobacteria	0.9	0.1	1.1	0.7	0.8	0.9	1.0	0.9	0.1	1.1	0.8	0.9	0.9	1.0
	Clostridia	31.9	0.9	33.0	29.9	31.6	32.0	32.5	30.4	3.6	34.3	26.1	27.2	30.6	33.8
	Coriobacteriia	0.0	0.0	0.1	0.0	0.0	0.0	0.0	0.0	0.0	0.1	0.0	0.0	0.0	0.0
	Deltaproteobacteria	1.2	0.5	1.9	0.6	0.7	1.0	1.6	1.2	0.1	1.4	1.1	1.2	1.2	1.3
	Gammaproteobacteria	21.9	1.4	23.6	19.2	21.1	21.9	22.9	17.8	5.3	24.4	12.1	12.5	17.9	23.0
	Not_Assigned	0.2	0.2	0.5	0.0	0.1	0.2	0.3	0.4	0.3	0.9	0.1	0.2	0.2	0.5
Order	Bacteroidales	43.7	2.1	46.9	41.2	41.8	43.7	45.0	49.0	1.9	51.2	46.1	47.3	48.9	50.9
	Bifidobacteriales	0.2	0.1	0.4	0.1	0.1	0.2	0.3	0.3	0.1	0.4	0.2	0.2	0.3	0.3
	Burkholderiales	0.9	0.1	1.1	0.7	0.8	0.9	1.0	0.9	0.1	1.1	0.8	0.9	0.9	1.0
	Clostridiales	31.9	0.9	33.0	29.9	31.6	32.0	32.5	30.4	3.6	34.3	26.1	27.2	30.6	33.8
	Coriobacteriales	0.0	0.0	0.1	0.0	0.0	0.0	0.0	0.0	0.0	0.1	0.0	0.0	0.0	0.0
	Desulfovibrionales	1.2	0.5	1.9	0.6	0.7	1.0	1.6	1.2	0.1	1.4	1.1	1.2	1.2	1.3
	Enterobacteriales	19.3	1.1	20.2	17.3	19.1	19.7	20.1	16.0	3.4	20.2	12.1	12.4	16.2	19.3
	Not_Assigned	0.2	0.2	0.5	0.0	0.1	0.2	0.3	0.4	0.3	0.9	0.1	0.2	0.2	0.5
	Xanthomonadales	2.5	0.7	4.1	1.9	2.0	2.3	2.9	1.9	2.0	4.6	0.0	0.0	1.7	3.5
Family	Alcaligenaceae	0.7	0.1	1.0	0.5	0.6	0.7	0.8	0.7	0.1	0.8	0.6	0.7	0.7	0.8
	Bacteroidaceae	29.9	1.9	32.9	27.3	28.5	30.0	31.0	32.3	1.7	34.4	29.3	31.1	32.5	33.6
	Bifidobacteriaceae	0.2	0.1	0.4	0.1	0.1	0.2	0.3	0.3	0.1	0.4	0.2	0.2	0.3	0.3
	Clostridiaceae	1.0	0.2	1.4	0.8	0.9	1.0	1.1	0.3	0.2	0.5	0.1	0.1	0.3	0.4
	Desulfovibrionaceae	1.2	0.5	1.9	0.6	0.7	1.0	1.6	1.2	0.1	1.4	1.1	1.2	1.2	1.3
	Enterobacteriaceae	19.3	1.1	20.2	17.3	19.1	19.7	20.1	16.0	3.4	20.2	12.1	12.4	16.2	19.3
	Lachnospiraceae	15.7	0.7	16.8	14.9	15.2	15.7	16.2	15.0	2.5	17.6	11.6	12.9	15.4	17.3

	Mogibacteriaceae	0.1	0.1	0.2	0.0	0.0	0.0	0.1	0.0	0.0	0.1	0.0	0.0	0.0	0.0
	Not_Assigned	1.4	0.2	1.9	1.2	1.3	1.4	1.5	2.5	0.5	3.2	1.7	2.0	2.6	2.8
	Odoribacteraceae	0.1	0.1	0.3	0.0	0.0	0.1	0.2	0.2	0.0	0.2	0.1	0.1	0.2	0.2
	Porphyromonadaceae	11.1	1.0	12.2	9.7	10.1	11.2	11.9	9.4	1.2	11.0	7.5	8.4	9.6	10.3
	Rikenellaceae	2.7	0.8	3.7	1.7	2.0	2.7	3.3	7.1	1.1	8.7	5.6	6.3	6.7	8.1
	Ruminococcaceae	7.8	1.0	9.5	6.9	7.1	7.4	8.5	7.6	1.0	8.9	6.1	6.8	7.3	8.4
	Veillonellaceae	6.3	1.6	8.4	4.4	4.8	6.2	7.6	5.6	1.7	7.6	3.5	3.9	5.7	7.2
	Xanthomonadaceae	2.5	0.7	4.1	1.9	2.0	2.3	2.9	1.9	2.0	4.6	0.0	0.0	1.7	3.5
Genus	Alistipes	1.8	0.4	2.4	1.3	1.5	1.8	2.1	5.7	0.9	7.1	4.3	5.2	5.3	6.4
	Bacteroides	29.9	1.9	32.9	27.3	28.5	30.0	31.0	32.3	1.7	34.4	29.3	31.1	32.5	33.6
	Bifidobacterium	0.2	0.1	0.4	0.1	0.1	0.2	0.3	0.3	0.1	0.4	0.2	0.2	0.3	0.3
	Bilophila	1.2	0.5	1.9	0.6	0.7	1.0	1.6	1.2	0.1	1.4	1.1	1.2	1.2	1.3
	Blautia	0.1	0.1	0.2	0.0	0.0	0.1	0.1	0.1	0.1	0.2	0.0	0.1	0.1	0.1
	Butyricicoccus	0.0	0.0	0.1	0.0	0.0	0.1	0.1	0.0	0.0	0.0	0.0	0.0	0.0	0.0
	Clostridium	3.4	0.2	3.8	3.1	3.3	3.4	3.5	5.8	1.8	7.8	3.5	4.2	6.0	7.4
	Collinsella	0.0	0.0	0.1	0.0	0.0	0.0	0.0	0.0	0.0	0.1	0.0	0.0	0.0	0.0
	Coprococcus	0.0	0.0	0.1	0.0	0.0	0.1	0.1	0.0	0.0	0.1	0.0	0.0	0.0	0.1
	Dialister	2.4	0.7	3.8	1.6	1.9	2.2	2.8	1.0	0.4	1.6	0.4	0.6	0.9	1.3
	Dorea	0.2	0.1	0.3	0.1	0.1	0.2	0.2	0.1	0.0	0.2	0.1	0.1	0.1	0.1
	Faecalibacterium	4.2	0.5	5.0	3.6	3.8	4.1	4.7	4.7	1.0	6.2	3.4	3.8	4.5	5.6
	Not_Assigned	39.7	1.1	40.9	37.4	39.3	40.2	40.5	29.2	2.5	32.9	26.0	27.1	29.5	31.0
	Odoribacter	0.1	0.1	0.3	0.0	0.0	0.1	0.2	0.2	0.0	0.2	0.1	0.1	0.2	0.2
	Oscillospira	1.7	0.2	1.9	1.5	1.7	1.8	1.9	1.4	0.1	1.6	1.1	1.3	1.3	1.4
	Parabacteroides	11.1	1.0	12.2	9.7	10.1	11.2	11.9	9.4	1.2	11.0	7.5	8.4	9.6	10.3
	Roseburia	0.1	0.0	0.1	0.0	0.1	0.1	0.1	0.1	0.1	0.3	0.0	0.1	0.1	0.2
	Ruminococcus	0.3	0.1	0.5	0.2	0.3	0.3	0.4	3.0	0.7	3.8	2.0	2.4	3.1	3.7
	Stenotrophomonas	2.5	0.7	4.1	1.9	2.0	2.3	2.9	1.9	2.0	4.6	0.0	0.0	1.7	3.5
	Sutterella	0.7	0.1	1.0	0.5	0.6	0.7	0.8	0.7	0.1	0.8	0.6	0.7	0.7	0.8
	Veillonella	0.3	0.1	0.4	0.2	0.2	0.3	0.3	2.8	0.4	3.5	2.0	2.5	2.8	3.1
